# Stereotype-based priming without stereotype activation: A tale of two priming tasks

**DOI:** 10.1177/1747021820925396

**Published:** 2020-07-07

**Authors:** Dimitra Tsamadi, Johanna K Falbén, Linn M Persson, Marius Golubickis, Siobhan Caughey, Betül Sahin, C Neil Macrae

**Affiliations:** 1 School of Psychology, University of Aberdeen, Aberdeen, UK; 2 School of Psychology, University of Plymouth, Plymouth, UK

**Keywords:** Stereotype activation, priming, response bias, automaticity, person perception

## Abstract

An extensive literature has demonstrated stereotype-based priming effects. What this work has only recently considered, however, is the extent to which priming is moderated by the adoption of different sequential-priming tasks and the attendant implications for theoretical treatments of person perception. In addition, the processes through which priming arises (i.e., stimulus and/or response biases) remain largely unspecified. Accordingly, here we explored the emergence and origin of stereotype-based priming using both semantic- and response-priming tasks. Corroborating previous research, a stereotype-based priming effect only emerged when a response-priming (vs. semantic-priming) task was used. A further hierarchical drift diffusion model analysis revealed that this effect was underpinned by differences in the evidential requirements of response generation (i.e., a response bias), such that less evidence was needed when generating stereotype-consistent compared with stereotype-inconsistent responses. Crucially, information uptake (i.e., stimulus bias, efficiency of target processing) was faster for stereotype-inconsistent than stereotype-consistent targets. This reveals that stereotype-based priming originated in a response bias rather than the automatic activation of stereotypes. The theoretical implications of these findings are considered.

A fundamental supposition in the psychological literature is that stereotypes simplify thinking and doing. Indeed, since Allport’s (1954) seminal writings, stereotypes are considered to economise social-cognitive functioning in an obligatory manner (Bargh, 1999; Bodenhausen & Macrae, 1998; Brewer, 1988; Devine, 1989; Fiske & Neuberg, 1990; Freeman & Ambady, 2011; but see Blair, 2002; Macrae & Bodenhausen, 2000). Grounded in the putative automaticity of stereotype activation, mere exposure to an individual is believed to trigger access to associated material in memory (i.e., stereotype contents) that shapes the course of interpersonal (and intergroup) exchanges. For example, preconceptions about an elderly dinner guest (e.g., conservative culinary preferences) may prompt the abandonment of one’s planned vindaloo for a less fiery alternative. Crucially, these stereotyped reactions are deemed to arise with neither people’s intention nor consent. But is this actually the case—is stereotype activation an inevitable product of the person-perception process (Allport, 1954; Bargh, 1999; Devine, 1989)? Consideration of the priming tasks commonly used to explore stereotype activation gives rise to a competing possibility (see Wentura & Degner, 2010; Wentura & Rothermund, 2014).

To investigate stereotype activation, sequential-priming procedures—whereby responses are facilitated when priming stimuli are followed by stereotype-consistent compared with stereotype-inconsistent targets—have been the predominant experimental tool (e.g., Banaji & Hardin, 1996; Blair & Banaji, 1996; Devine, 1989; Dovidio et al., 1986; Kawakami & Dovidio, 2001; Macrae & Cloutier, 2009; Macrae & Martin, 2007; Perdue & Gurtman, 1990). These priming tasks come in two varieties: response- and semantic-priming paradigms (see Wentura & Degner, 2010; Wentura & Rothermund, 2014). Although regarded to be equivalent in many accounts of stereotype-based priming (Bargh, 1999; Blair, 2002; Macrae & Bodenhausen, 2000), this is not the case. Indeed, as the cognitive origins of priming differ across these tasks, particular patterns of results have important implications for extant theoretical treatments of person construal and the automaticity of stereotype activation (Bargh, 1999; Blair, 2002; Bodenhausen & Macrae, 1998; Brewer, 1988; Devine, 1989; Fiske & Neuberg, 1990; Freeman & Ambady, 2011; Macrae & Bodenhausen, 2000; Wentura & Rothermund, 2014).

In response-priming tasks, participants are required to categorise target stimuli along a stereotype-relevant dimension. For example, in a prominent article, Blair and Banaji (1996) demonstrated that forenames (e.g., *Alice*) were categorised faster according to sex (i.e., male or female) when they were preceded by gender-congruent (e.g., *sensitive*) compared with gender-incongruent (e.g., *decisive*) attributes. Underlying effects of this kind is the assumption that, following presentation of a priming stimulus, activation spreads to semantically associated information in memory, thereby facilitating responses to related (vs. unrelated) material (Collins & Loftus, 1975; Neely, 1991).^1^ It should be noted, however, that verbal priming procedures are poorly suited to explore stereotype activation during person perception as, ironically, no actual persons are presented to participants in the course of the task (Macrae & Bodenhausen, 2000). Instead, a better approach is to utilise facial primes as triggering categorical stimuli. For instance, following the presentation of male or female faces, participants must report if gender-related targets (e.g., *cigar, flowers*) are stereotypically masculine or feminine given prevailing societal beliefs about the sexes (Castelli et al., 2004; Kawakami & Dovidio, 2001; Macrae & Cloutier, 2009; Macrae & Martin, 2007; Müller & Rothermund, 2014). In contrast, in semantic-priming procedures, responses are irrelevant to the stereotypic dimension under investigation. Using a Lexical Decision Task (LDT), for example, participants must state if stereotype-related targets comprise words or nonwords (Casper et al., 2010, 2011; Macrae et al., 2002; Sassenberg & Moskowitz, 2005; Wittenbrink et al., 2001).

Interestingly, these divergent sequential-priming tasks have produced mixed results in investigations of stereotype-based priming (K. R. G. White et al., 2018). As revealed in recent meta-analytic work (Kidder et al., 2018), whereas significant priming effects consistently arise in response-priming tasks, this is not the case when semantic-priming methodologies are employed. For example, a non-significant priming effect has been observed when stereotype activation is probed using an LDT. Driving this inconsistency is likely differences in the cognitive operations that underpin response- and semantic-priming, respectively (Wentura & Degner, 2010; Wentura & Rothermund, 2014). Although spreading activation provides a viable explanation for priming effects in semantic-priming tasks (i.e., primes pre-activate related concepts in memory, thereby facilitating target processing and task performance), matters are more complicated when response-priming procedures are adopted, the very methodologies under which stereotype-based priming is reliably observed (Kidder et al., 2018). In these tasks, as with semantic-priming paradigms, response facilitation can be attributed to enhanced target processing (via spreading activation). In addition, however, as the judgement rendered on the target stimuli is also applicable to the primes, priming can be underpinned by a quite different mechanism—response facilitation/competition (Wentura & Degner, 2010). Specifically, prior to target presentation, exposure to the prime triggers the generation of a compatible or incompatible response, such that performance is enhanced when prime and target elicit congruent (vs. incongruent) reactions. In this way, priming can be driven by both spreading activation and/or response generation, a state of affairs that may explain why stereotype-based priming is more likely to emerge in response-priming than semantic-priming tasks. For this reason, in the current investigation we sought to identify the pathway (or pathways) through which stereotype-based priming arises in response-priming tasks.

Identifying the origin of stereotype-based priming has important implications for theoretical accounts of person perception and stereotype automaticity (Kidder et al., 2018; Wentura & Degner, 2010; Wentura & Rothermund, 2014; K. R. G. White et al., 2018). If, for example, priming is driven exclusively by response-related processes (vs. spreading activation), then this would undermine the assumption that exposure to a person triggers obligatory access to stereotype-related material in memory (i.e., stereotype-based priming is not underpinned by stereotype activation), a belief that has dominated thinking on this topic for decades (Allport, 1954; Bargh, 1999; Brewer, 1988; Devine, 1989; Fiske & Neuberg, 1990; Freeman & Ambady, 2011). If, however, priming is underpinned by spreading activation this would provide evidence for the automaticity of stereotype activation. What is needed, therefore, is a means through which the operations that underpin stereotype-based priming can be identified. Usefully, a drift diffusion model analysis performs just such a function (Voss, Rothermund, et al., 2013). Applied successfully across a range of domains (see Wagenmakers, 2009), the drift diffusion model uses both response accuracy and latency to represent the decision-making process as it unfolds over time, thereby enabling the latent cognitive operations associated with decisional processing to be estimated (Ratcliff, 1978; Ratcliff et al., 2016; Ratcliff & Rouder, 1998; Voss et al., 2015; Voss, Nagler, & Lerche, 2013; Voss & Voss, 2007).

During binary decision-making (e.g., is a target stereotypically masculine or feminine?), information is continuously gathered from a stimulus until sufficient evidence is acquired to make a response (e.g., the target is feminine). As noted previously, in such a decisional context, stereotype-based priming may emerge via cognitive pathways pertaining to the efficiency of target processing and/or the generation of compatible/incompatible target-related responses (i.e., stimulus and/or response biases, C. N. White & Poldrack, 2014). Specifically, stereotype-consistent targets may be identified more rapidly than their stereotype-inconsistent counterparts because (1) primes facilitate information uptake for prime-congruent compared with prime-incongruent stimuli (i.e., stimulus bias); and/or (2) primes modulate information-sampling requirements (i.e., response bias), such that less evidence is needed to generate prime-congruent than prime-incongruent responses. Importantly, these biases map onto the processes that underpin task performance in response-priming tasks (Wentura & Degner, 2010; Wentura & Rothermund, 2014), thereby yield potentially valuable insights into the origins of stereotype-based priming. Whereas spreading activation is indexed by the rate of evidence accumulation during target processing (Voss, Rothermund, et al., 2013), a response bias is captured by the evidential requirements of response generation (Dunovan et al., 2014; C. N. White & Poldrack, 2014).

Extending previous research on this theoretically important topic (K. R. G. White et al., 2018), here we explored stereotype-based priming using a Stereotype Classification Task (SCT—response-priming paradigm). Adopting a standard sequential-priming methodology, following the presentation of male or female faces (i.e., primes), participants responded to targets (i.e., object labels) that were either consistent or inconsistent with respect to prevailing stereotypes about the sexes (Blair & Banaji, 1996; Castelli et al., 2004; Macrae & Martin, 2007; Mason et al., 2006). To decompose decisional processing and identify the origin of stereotype-based priming, data were submitted to a Hierarchical Drift Diffusion Model (HDDM) analysis (Wiecki et al., 2013). Although meta-analytic work has revealed that semantic-priming paradigms are likely to yield a null effect (Kidder et al., 2018), we also used an LDT to probe stereotype-based priming. As it is possible that stereotype-based priming effects in a response-priming task are underpinned by spreading activation, an HDDM analysis may be sensitive enough to uncover this effect in an LDT. Accordingly, whereas half of our participants reported whether target stimuli were stereotypically feminine or masculine in implication (i.e., SCT), the others judged the lexical status (i.e., word or nonword) of the items (i.e., LDT).

## Method

### Participants and design

Seventy participants (25 males, *M*_age_ = 22.24, *SD* = 2.32) took part in the experiment, 36 in the SCT and 34 in the LDT. Three participants failed to follow the instructions, thus were excluded from the analyses (3 participants from the SCT). To replicate the established stereotype-based priming effect using a SCT, our sample size was based on the meta-analytic effect size reported by Kidder et al. (2018), such that G*Power (*d* *=* .52, α = .05, power = 80%) indicated a requirement of 32 participants (an additional 10% were recruited to allow for drop out). A comparable number of participants completed the LDT. Informed consent was obtained from participants prior to the commencement of the experiment and the protocol was reviewed and approved by the Ethics Committee at the School of Psychology, University of Aberdeen. The experiment had a 2 (Task: SCT or LDT) × 2 (Prime: female or male) × 2 (Target: feminine or masculine) mixed design, with repeated measures on the second and third factors.

### Stimulus materials and procedure

Participants arrived at the laboratory individually, were greeted by the experimenter, seated in front of a desktop computer, and told they would be performing a word-classification task. They were then randomly allocated to perform either the SCT or the LDT. In the SCT, following the presentation of a male or a female face, participants had to report, using two buttons on the keyboard, whether an object label was feminine (i.e., *perfume, doll, flower, dress, & lipstick*) or masculine (i.e., *beer, hammer, bowtie, briefcase, & cigar*) in implication, given prevailing gender stereotypes. Participants initially performed 12 practice trials, followed by five blocks of 120 experimental trials in which stereotype-consistent (i.e., female face/feminine object or male face/masculine object) and stereotype-inconsistent (i.e., female face/masculine object or male face/feminine object) stimuli appeared equally often in a random order.

In the LDT, following the presentation of a male or a female face, participants had to report, using two buttons on the keyboard, whether the presented stimulus was a word (see above) or a nonword (i.e., *feumper, lodl, rowfel, serds, cliptiks, reeb, remham, owtbie, acrefibes, & cargi*). Nonwords were constructed from the words by shuffling the letters to produce pronounceable stimuli. Participants initially performed 16 practice trials, followed by five blocks of 240 experimental trials, of which 120 comprised word stimuli and 120 nonword stimuli. As in the SCT, half of the word trials comprised stereotype-consistent pairings and half comprised stereotype-inconsistent pairings. All stimulus pairings appeared equally often in a random order.

In both tasks, each trial began with the presentation of a central fixation cross for 500 ms, followed by a face (i.e., female or male), which remained on the screen for 250 ms, after which it disappeared and was replaced by an object label in the SCT and for half of the trials in the LDT, or a nonword for the remaining trials in the LDT, for 1,000 ms. Participants had 1,500 ms to make a response and the inter-trial interval was 500 ms. The meaning of the response buttons (i.e., N & M) was counterbalanced across participants for both tasks. Primes (30 female & 30 male faces) were taken from the Chicago Face Database (Ma et al., 2015) and were 140 × 176 pixels in size, greyscale, and depicted young Caucasian adults aged 20–30 years. Target words were taken from Crawford et al. (2004). On completion of the experiment, participants were debriefed, thanked, and dismissed.

## Results

### Response time

Analyses were undertaken on participants’ correct responses. Responses faster than 200 ms were excluded from the analyses, eliminating approximately 2% of the overall number of trials (see Table 1 for treatment means).^2^ A multilevel model analysis was used to examine the response time (RT) data. Analyses were conducted with the R package “lmer4” (Pinheiro et al., 2015). Task, Prime, and Target were treated as categorical fixed effects, and participants as a crossed-random effect (Judd et al., 2012). The analysis yielded several significant main effects and interactions, including the critical Task × Prime × Target interaction (*b* = .028, *SE* = .005, *t* = 5.62, *p* < .001; see Supplementary Material for a complete listing of the results). To further explore the three-way interaction, separate 2 (Prime: female or male) × 2 (Target: feminine or masculine) multilevel analyses were conducted for each Task. For the SCT, the analysis yielded a significant Prime × Target interaction (*b* = −.024, *SE* = .004, *t* = −6.34, *p* < .001). Further analysis of the interaction revealed that, when targets were feminine, responses were faster when they were primed with female compared with male faces (*b* = −.010, *SE* = .003, *t* = −3.56, *p* < .001). In contrast, when targets were masculine, responses were faster when they were primed with male than female faces (*b* = .015, *SE* = .003, *t* = 5.50, *p* < .001). For the LDT, no significant Prime × Target interaction was observed (*b* = .004, *SE* = .003, *t* = 1.20, *p* = .231).

**Table 1.** Response time (ms) and accuracy (%) as a function of task, prime, and target.

	Prime			
	Female		Male	
Target	Feminine	Masculine	Feminine	Masculine
Task				
SCT				
RT	562 (71)	578 (68)	572 (66)	562 (71)
Accuracy	91 (7)	89 (11)	89 (10)	92 (7)
LDT				
RT	549 (53)	566 (56)	551 (49)	572 (53)
Accuracy	97 (2)	93 (5)	97 (3)	94 (6)

SCT: Stereotype Classification Task; RT: response time; LDT: Lexical Decision Task.

Standard deviation (*SD*) in parentheses.

### Accuracy

A multilevel logistic regression analysis on the accuracy of participants’ responses revealed a significant Task × Prime × Target interaction (*b* = −.527, *SE* = .176, *z* = −2.99, *p* = .003; see Supplementary Material for a complete listing of the results). To further explore the three-way interaction, separate 2 (Prime: female or male) × 2 (Target: feminine or masculine) multilevel analyses were conducted for each Task. For the SCT, this yielded a significant Prime × Target interaction (*b* = .668, *SE* = .105, *z* = 6.36, *p* < .001). Further analysis of the interaction revealed that, when targets were feminine, responses were more accurate when they were primed with female compared with male faces (*b* = .271, *SE* = .073, *z* = 3.69, *p* < .001). In contrast, when targets were masculine, responses were more accurate when they were primed with male than female faces (*b* = −.401, *SE* = .075, *t* = −5.33, *p* < .001). For the LDT, no Prime × Target interaction was observed (*b* = .141, *SE* = .141, *z* = 1.00, *p* = .319).

### Diffusion modelling

To identify the processes underpinning the significant stereotype-based priming effect observed in the SCT, data were submitted to an HDDM analysis (Wiecki et al., 2013).^3^ HDDM is an open-source software package written in Python for the hierarchical Bayesian estimation of drift diffusion model parameters. This approach assumes that the model parameters for individual participants are random samples drawn from group-level distributions and uses Bayesian statistical methods to estimate all parameters at both the group- and individual-participant level (Vandekerckhove et al., 2011). The drift diffusion model asserts that, during binary decision-making, noisy information is sequentially sampled until sufficient evidence is acquired to make a response (Ratcliff, 1978; Ratcliff et al., 2016; Voss et al., 2015).

The duration of the diffusion process is known as the decision time, and the process can be characterised by several important parameters. Drift rate (*v*) estimates the speed of information gathering (i.e., larger drift rate = faster information uptake), thus is interpreted as a measure of the efficiency of stimulus processing during decision-making. Boundary separation (*a*) estimates the distance between the two decision thresholds (e.g., feminine vs. masculine), hence indicates how much evidence is required before a response is made (i.e., larger [smaller] values indicate more conservative [liberal] responding). The starting point (*z*) defines the position between the decision thresholds at which evidence accumulation begins. If *z* is not centred between the thresholds (*z* ≠ .50), this denotes an a priori bias in favour of the response that is closer to the starting point (i.e., response-expectancy bias). In other words, less evidence is required to reach the preferred (vs. non-preferred) threshold. Finally, the duration of all non-decisional processes is given by the additional parameter *t_0_*, which is taken to indicate differences in stimulus encoding and response execution.

HDDM is useful in the current investigation as it decomposes task performance (i.e., RTs & accuracy) into the latent psychological operations that underpin task performance—notably, speed/efficiency of stimulus processing (i.e., stimulus bias—drift rate *v*), information-sampling requirements (i.e., response bias—starting value *z*), and non-decisional processes (*t*_0_)—thereby revealing the origin of stereotype-based priming effects. If stereotype-based priming is underpinned by a stimulus bias (i.e., spreading activation), one would expect the drift rate (*v*) to be larger for stereotype-consistent than stereotype-inconsistent items. In contrast, if priming is underpinned by a response bias (i.e., shift in the starting point, *z*), one would expect less evidence to be required to generate stereotype-consistent compared with stereotype-inconsistent responses.

Models were response coded, such that the upper threshold corresponded to feminine responses and the lower threshold to masculine responses. Bayesian posterior distributions were modelled using a Markov Chain Monte Carlo (MCMC) with 10,000 samples (following 1,000 burn in samples). Outliers (5% of trials) were removed by the HDDM software (Ratcliff & Tuerlinckx, 2002). Eight models were estimated for comparison (see Table 2). In model 1, only drift rate was allowed to vary as a function of Target. In four models, we investigated whether there was a bias in the evidential requirements of response generation as a function of the Prime (i.e., response bias). That is, although all models included *z* as a free parameter, in models 2, 4, 6, and 8 two different *z* parameters were estimated for trials with male and female primes. Next, in four models, we considered whether there was a bias in the efficiency of stimulus processing (models 3, 4, 7, & 8). Finally, four combinations of non-decision time (*t_0_*) were allowed to vary across Prime (i.e., female or male) and Target (i.e., feminine or masculine; models 5–8). Across all models, drift rate (*v*) was allowed to vary as a function of Target to establish if the speed of information uptake was equivalent for feminine and masculine targets. The estimated model values were positive for feminine targets and negative for masculine targets. Values for masculine targets were sign-reversed, such that absolute drift rates were compared. Boundary separation (*a*) and inter-trial variability in starting point (*s_z_*), drift rate (*s_v_*), and non-decision time (*s_t0_*) were held constant across trials to increase model parsimony and fit (Voss, Nagler, & Lerche, 2013).

**Table 2.** Model comparison (deviance information criterion) for the SCT.

Allowed to vary by			
Model	Prime	Target	DIC
1.	*–*	*v*	−19,796
2.	*z*	*v*	−20,064
3.	*v*	*v*	−19,862
4.	*z, v*	*v*	−20,059
5.	*t_0_*	*v, t_0_*	−19,941
6.	*z, t_0_*	*v, t_0_*	−20,118
7.	*v, t_0_*	*v, t_0_*	−20,001
8.	*z, v, t_0_*	*v, t_0_*	−20,124

SCT: Stereotype Classification Task; DIC: deviance information criterion; *v*: drift rate; *z*: starting point; *t_0_*: non-decision time.

A DIC difference of 5 is strong evidence for a model (Tipples, 2018).

As can be seen from Table 2, model 8 yielded the best fit (i.e., lowest Deviance Information Criterion [DIC] value). The DIC was adopted as it is routinely used for hierarchical Bayesian model comparison (Spiegelhalter et al., 1998). As diffusion models were fit hierarchically rather than individually for each participant, a single value was calculated for each model that reflected the overall fit to the data at the participant- and group-level. Lower DIC values favour models with the highest likelihood and least number of parameters.

**Figure 1.**
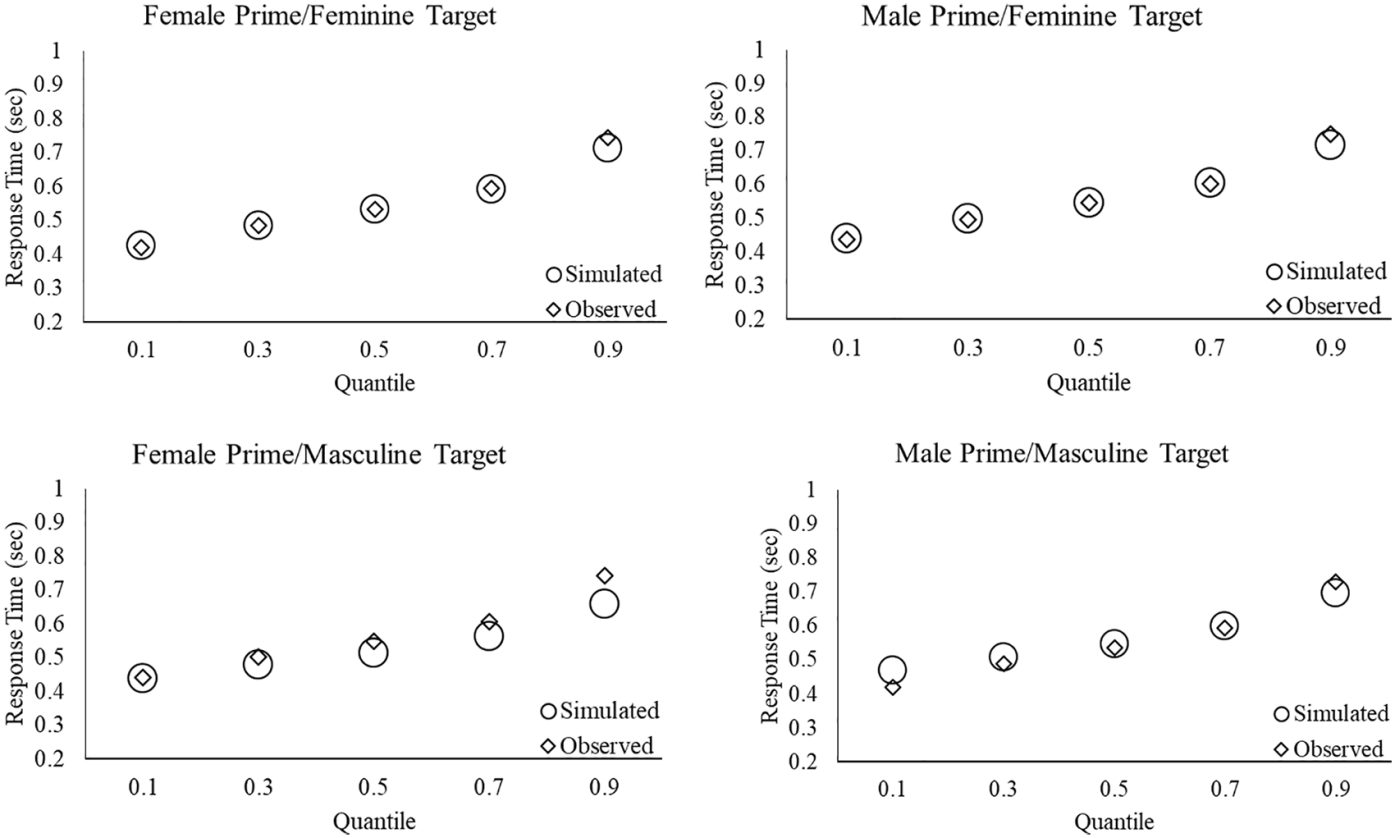
Posterior Predictive Check. Comparison of simulated data generated by the best fitting model (i.e., model 8) and observed data for each experimental condition for the .1, .3, .5, .7, and .9 RT quantiles.

As a graphical approach to assess goodness-of-fit, a standard model procedure used in Bayesian parameter estimation—Posterior Predictive Check (PPC)—was performed (Wiecki et al., 2013). For model 8, the posterior distributions of the estimated parameters were used to simulate data sets. We then assessed the quality of model fit by plotting the observed data against the simulated data for the .1, .3, .5, .7, and .9 response time quantiles for each experimental condition (Falbén et al., 2019; Krypotos et al., 2015). As can be seen in Figure 1, with nearly complete overlap between the observed values and the simulated estimates across all prime-target combinations, this revealed good model fit.

Interrogation of the posterior distributions for model 8 revealed that task performance was underpinned by a combination of response and stimulus biases (see Table 3). Comparison of the observed starting values (female prime: *z* = .53; male prime: *z* = .45) with no bias (*z* = .50) yielded strong evidence that less information was required when making stereotype-consistent than stereotype-inconsistent responses, following both female, *p*_Bayes_(bias > .50) = .001, and male, *p*_Bayes_(bias < .50) < .001, primes.^4^ In addition, moderate evidence for a stimulus bias was also observed, indicating that information uptake was faster for stereotype-inconsistent compared with stereotype-consistent material for feminine targets, *p*_Bayes_(female prime/feminine target < male prime/feminine target) = .118. For masculine targets, only suggestive evidence for this stimulus bias emerged, *p*_Bayes_(male prime/masculine target < female prime/masculine target) = .239.^5^ No differences were observed in the speed of non-decisional processes (*t_0_*).

**Table 3.** Parameter means and 95% highest density intervals (HDI) of the best fitting model for the SCT.

Model parameter	Mean	95% HDI	
		Lower threshold	Upper threshold
*a*	1.057	0.988	1.132
*v*_Female Prime/Feminine Target_	3.251	2.839	3.661
*v*_Female Prime/Masculine Target_	−3.563	−3.972	−3.161
*v*_Male Prime/Feminine Target_	3.585	3.172	3.998
*v*_Male Prime/Masculine Target_	−3.385	−3.814	−3.001
*z*_Female Prime_	0.531	0.512	0.551
*z*_Male Prime_	0.453	0.433	0.473
*t_0_*_Female Prime/Feminine Target_	0.417	0.340	0.434
*t_0_*_Female Prime/Masculine Target_	0.423	0.405	0.441
*t_0_*_Male Prime/Feminine Target_	0.417	0.399	0.434
*t_0_*_Male Prime/Masculine Target_	0.425	0.407	0.443
s_v_	0.795	0.573	1.003
s_z_	0.547	0.495	0.598
*s*_t0_	0.160	0.155	0.165

HDI: highest density intervals; SCT: Stereotype Classification Task; *a*: boundary separation; *v*: drift rate; *z*: starting point; *t_0_*: non-decision time; *s_v_*: inter-trial variability of drift rate; *s_z_*: inter-trial variability of starting point; *s_t0_*: inter-trial variability in non-decision time.

## Discussion

Using both semantic- and response-priming paradigms, here we explored the origins of stereotype-based priming. Corroborating previous behavioural and meta-analytic work, a priming effect only emerged when a response-priming (vs. semantic-priming) task was used (Kidder et al., 2018; Müller & Rothermund, 2014; K. R. G. White et al., 2018). In addition, a further HDDM analysis revealed that this effect was underpinned by differences in the evidential requirements of response generation, such that less evidence was needed when generating stereotype-consistent compared with stereotype-inconsistent responses. Interestingly, the efficiency of stimulus processing did not contribute to the emergence of this priming effect. Instead, information uptake was faster for stereotype-inconsistent than stereotype-consistent targets. What this then reveals is that, in response-priming tasks, stereotype-based priming originates in the generation of a response bias and not the enhanced processing of stereotype-consistent (vs. stereotype-inconsistent) stimuli (Wentura & Degner, 2010; Wentura & Rothermund, 2014).^6^ In other words, stereotype-based priming is not underpinned by stereotype activation.

The current findings have important implications for theoretical treatments of stereotype automaticity (Bargh, 1999; Blair, 2002; Devine, 1989; Fiske & Neuberg, 1990; Macrae & Bodenhausen, 2000). Based on the adoption of sequential-priming paradigms, researchers have concluded that stereotypes are automatically activated following the presentation of triggering category-related primes. Specifically, primes facilitate the processing of stereotype-consistent compared with stereotype-inconsistent stimuli (Banaji & Hardin, 1996; Blair & Banaji, 1996; Devine, 1989; Dovidio et al., 1986; Kawakami & Dovidio, 2001; Macrae & Cloutier, 2009; Macrae & Martin, 2007; Perdue & Gurtman, 1990). Critically, however, many of the studies that purport to demonstrate the automaticity of stereotype activation have used response-priming tasks in which the origins of priming potentially reside in response-related processes (e.g., Blair & Banaji, 1996; Kawakami et al., 2000; Macrae et al., 2002; Macrae & Cloutier, 2009; Macrae & Martin, 2007). Substantiating this concern, in a response-priming task, here we demonstrated that stereotype-based priming was underpinned by a response bias and not the enhanced processing of stereotype-related material. Moreover, in a task well equipped to explore the automaticity of stereotype activation (i.e., LDT), no priming effect was observed (Kidder et al., 2018; Müller & Rothermund, 2014; K. R. G. White et al., 2018). These findings affirm that consideration should be given to the manner in which stereotype-based priming is probed before assertions about the automaticity of stereotype activation are advanced (Wentura & Degner, 2010; Wentura & Rothermund, 2014). Based on the current findings, it appears that an extensive literature purporting to demonstrate the automaticity of stereotype activation in reality may reveal no such thing (Bargh, 1999; Brewer, 1988; Devine, 1989; Fiske & Neuberg, 1990; Freeman & Ambady, 2011). Instead, stereotype-based priming is underpinned by prime-target response compatibility.

Of additional theoretical interest, although a stimulus bias was observed in the current experiment, this did not contribute to stereotype-based priming. Rather, primes enhanced the processing of stereotype-inconsistent compared with stereotype-consistent targets. Albeit using a different experimental paradigm (i.e., explicit face-label stereotype-based judgement task), Falbén et al. (2019) recently reported an identical effect. To guide social-cognitive functioning in an adaptable manner, the mind must possess two complementary skills (see Grossberg, 1987; Johnston & Hawley, 1994; McClelland et al., 1995). First, it must sensitise people to invariant aspects of the world (i.e., need for stability). Second, it must be responsive to the presence of unexpected stimulus inputs (i.e., need for plasticity). As uncovered by the HDDM analysis, both of these effects were observed in the current inquiry, with each bias underpinned by a different cognitive mechanism. Whereas the mind’s responsivity towards expected (vs. unexpected) inputs was reflected in the operation of a response bias (i.e., starting point, *z*), decisional evidence was accumulated more rapidly for stereotype-inconsistent (vs. stereotype-consistent) targets (i.e., drift rate, *v*), indicating sensitivity towards unexpected inputs. Again, at least in the context of a response-priming task, this challenges the widely held assumption that stereotypes facilitate the processing of stereotype-consistent (vs. stereotype-inconsistent) material (Bargh, 1999; Brewer, 1988; Devine, 1989; Dovidio et al., 1986; Fiske & Neuberg, 1990; Perdue & Gurtman, 1990).

Together with related research, the current results speak to the task-dependent nature of stereotype-based priming. Elsewhere, stereotype activation has failed to emerge when people are demotivated, attentionally challenged, or instructed to process priming stimuli on the basis of low-level perceptual processing objectives (Blair, 2002; Macrae & Bodenhausen, 2000). For example, Macrae et al. (1997) showed that stereotype activation was eliminated when participants performed a visual search task (i.e., dot detection) on facial primes (see also Wheeler & Fiske, 2005). As Bruner (1957, p. 132) famously noted, “The accessibility of categories I employ for identifying the objects of the world around me . . . must reflect the search requirements imposed by my needs, my ongoing activities, my defences etc.” As an emerging literature is disclosing, stereotype activation is also contingent on the manner in which priming is measured (Kidder et al., 2018; Müller & Rothermund, 2014; Wentura & Degner, 2010; Wentura & Rothermund, 2014; K. R. G. White et al., 2018). Notably, priming is most likely to emerge when response- rather than semantic-priming tasks are used.

Of course, the failure to observe stereotype-based priming when using semantic-priming tasks—especially an LDT—does not mean that such effects never arise. It is possible that the LDT simply lacks the sensitivity to detect delicate stereotype-based priming effects in certain task contexts. For example, using an LDT, Casper and colleagues reported stereotype-based priming when verbal primes were presented in combination with matching pictorial contexts (Casper et al., 2010, 2011; Casper & Rothermund, 2012). In addition, other semantic-priming tasks (e.g., word pronunciation, semantic classification) can also be used to explore stereotype-based priming, with spreading activation potentially contributing to the emergence of priming effects (see Kidder et al., 2018). In related investigations of evaluative priming, for example, the involuntary activation of a prime’s valence has been shown to depend on the goal state that is operating and the proportion of stimuli with distinct affective connotations (e.g., Everaert et al., 2011; Spruyt, De Houwer, et al., 2007; Spruyt, Hermans, et al., 2007). A useful task for future research will therefore be to identify the task-related factors that influence the emergence and origin of stereotype-based priming. Work of this kind is important for both methodological and theoretical reasons. For over six decades, it has been assumed that stereotypes are automatically activated in the presence of group members (Allport, 1954; Bargh, 1999; Devine, 1989; Fiske & Neuberg, 1990). Using the sophisticated experimental and computational tools that are readily available to researchers (Ratcliff et al., 2016; Voss et al., 2015; Wentura & Degner, 2010; Wiecki et al., 2013), it is now time to establish exactly when, why, and for whom this may (or may not) be the case.

## Supplemental Material

QJE-STD-19-245.R3-Supplementary_Material – Supplemental material for Stereotype-based priming without stereotype activation: A tale of two priming tasks Click here for additional data file. Supplemental material, QJE-STD-19-245.R3-Supplementary_Material for Stereotype-based priming without stereotype activation: A tale of two priming tasks by Dimitra Tsamadi, Johanna K Falbén, Linn M Persson, Marius Golubickis, Siobhan Caughey, Betül Sahin and C Neil Macrae in Quarterly Journal of Experimental Psychology

## Notes

1. A competing account suggests that priming is underpinned by an overlap between prime and target representations in semantic memory, such that processing the prime and the target triggers similar patterns of activation (Masson, 1995; Rumelhart et al., 1986). This viewpoint, however, has not been widely advanced as an explanation for stereotype-based priming (Freeman & Ambady, 2011).

2. In the LDT, only the word trials were submitted to statistical analysis.

3. Although the LDT did not yield a significant stereotype-based priming effect, a drift diffusion model analysis was conducted on these data (see Supplementary Material).

4. Bayesian *p* values quantify the degree to which the difference in the posterior distribution is consistent with the hypothesis that the parameter is greater for consistent than inconsistent responses. For example, a Bayesian *p* of .05 indicates that 95% of the posterior distribution supports the hypothesis (Kruschke, 2010; Lee & Wagenmakers, 2013).

5. To rule out the possibility that this incongruence effect may be a methodological artefact based on the influence of fast responses, which have a particularly strong influence on parameter estimation, we conducted additional HDDM analyses using stricter outlier criteria (i.e., responses faster than 300 ms and 350 ms were removed). In each of these analyses, the results were unchanged (i.e., an incongruence effect on drift rates was observed).

6. Using *fast-dm*, Voss, Rothermund, et al. (2013) traced priming effects in a response-priming task to differences in non-decisional processes (*t_0_*), such that response execution was faster following congruent than incongruent primes. Importantly, however, it was not possible to estimate a model with a free starting point (*z*) in this investigation. As reported here, when both *z* and *t_0_* were estimated simultaneously, priming effects originated in the pre-selection of compatible (vs. incompatible) prime-target responses.
