# Supplementary material for: Stereotype-based priming without stereotype activation: A tale of two priming tasks
Source: Q J Exp Psychol (Hove). 2020 Jul 7;73(11):1939–48. doi: 10.1177/1747021820925396 (PMC7586007; doi:10.1177/1747021820925396)
Supplement: QJE-STD-19-245.R3-Supplementary_Material – Supplemental material for Stereotype-based priming without stereotype activation: A tale of two priming tasks [file QJE-STD-19-245.R3-Supplementary_Material.docx]

Supplementary Material for:

**Stereotype-Based Priming Without Stereotype Activation:**

**A Tale of Two Priming Tasks**

Dimitra Tsamadi, Johanna K. Falbén, Linn M. Persson, Marius Golubickis, Siobhan Caughey, Betül Sahin, C. Neil Macrae

**Multi-Level Modeling (MLM) Tables**

**Combined Analysis**

## MLM – Response Time (Fixed effects)

**b Std. Error df t p**

Intercept 0.562 0.063 68 52.897 < .001

Prime 0.015 0.003 37085 5.763 < .001

Target 0.009 0.003 37085 3.753 < .001

Task 0.010 0.015 68 0.667 .507

Prime * Target -0.024 0.004 37085 -6.699 < .001

Prime * Task -0.046 0.004 37085 -5.729 < .001

Target * Task -0.031 0.004 37085 -8.699 < .001

Prime * Target * Task 0.028 0.005 37085 5.621 < .001

**SCT**

**MLM – Response Time (Fixed effects)**

**b Std. Error df t p**

Intercept 0.562 0.012 343.00 47.595 < .001

Prime 0.015 0.003 17770 5.458 < .001

Target 0.010 0.003 17770 3.554 < .001

Prime * Target -0.024 0.004 17770 -6.345 < .001

**LDT**

**MLM – Response Time (Fixed effects)**

**b Std. Error df t p**

Intercept 0.572 0.009 35.880 64.668 < .001

Prime -0.006 0.002 19320 -2.417 .016

Target -0.021 0.002 19320 -9.149 < .001

Prime * Target 0.004 0.003 19320 1.199 .231

**Combined Analysis**

**MLM- Accuracy (Fixed effects)**

**b Std. Error z p**

Intercept 2.810 0.147 19.165 < .001

Prime -0.397 0.075 -5.289 < .001

Target -0.384 0.075 -5.097 < .001

Task 0.127 0.207 0.613 .540

Prime * Target 0.668 0.105 6.355 < .001

Prime * Task 0.319 0.111 2.883 .004

Target * Task 1.143 0.125 9.152 < .001

Prime * Target * Task -0.527 0.176 -2.991 .003

**SCT**

**MLM – Accuracy (Fixed effects)**

**b Std. Error z p**

Intercept 2.810 0.147 19.134 < .001

Prime -0.397 0.075 -5.293 < .001

Target -0.384 0.075 -5.102 < .001

Prime * Target 0.668 0.105 6.359 < .001

**LDT**

**MLM – Accuracy (Fixed effects)**

**b Std. Error z p**

Intercept 2.936 0.146 20.126 < .001

Prime -0.078 0.081 -0.962 .336

Target 0.759 0.100 7.623 < .001

Prime * Target 0.141 0.141 0.996 .319

**Hierarchical Drift Diffusion Modeling of LDT Data**

An additional HDDM analysis was conducted to explore performance during the LDT. Models were response coded, such that the upper threshold corresponded to responses to words and the lower threshold to responses to nonwords. Bayesian posterior distributions were modeled using a Markov Chain Monte Carlo (MCMC) with 10,000 samples (following 1,000 burn in samples). Outliers (5% of trials) were removed by the HDDM software (Ratcliff & Tuerlinckx, 2002).

Eight models were estimated for comparison (see Table S1). In model 1, only drift rate was allowed to vary as a function of Target (i.e., word or nonword) and Target Type (i.e., feminine, masculine or nonword). In four models, we investigated whether there was a bias in the evidential requirements of response generation as a function of the Prime (i.e., female or male, response bias). That is, although all models included *z* as a free parameter, in models 2, 4, 6, and 8 two different *z* parameters were estimated for trials with male and female primes. Next, in four models, we considered whether there was a bias in the efficiency of stimulus processing (models 3, 4, 7, & 8). Finally, four combinations of non-decision time (*t_0_*) were allowed to vary across Prime, Target, and Target Type (i.e., models 5-8). Across all models, drift rate (*v*) was allowed to vary as a function Target and Target Type to establish if the speed of information uptake was equivalent for feminine and masculine words and nonwords. The estimated model values were positive for words and negative for nonwords. Values for nonwords were sign-reversed, such that absolute drift rates were compared. Boundary separation (*a*) and inter-trial variability in starting point (*s_z_*), drift rate (*s_v_*), and non-decision time (*s_t0_*) were held constant across trials to increase model parsimony and fit (Voss, Nagler, & Lerche, 2013). As can be seen from Table S1, model 6 yielded the best fit (i.e., lowest Deviance Information Criterion [DIC] value).

*Table S1.* Model comparison (Deviance Information Criterion) for the LDT.

Allowed to vary by

Model Prime Target Target Type DIC

1. *-* *v v* -60636

2. *z v v* -60623

3. *v v v* -60547

4. *z, v v v* -60557

5. *t_0_ v, t_0_ v, t_0_*  -60943

6. *z, t_0_ v, t_0_ v, t_0_* -60947

7. *v, t_0_ v, t_0_ v, t_0_* -60895

8. *z, v, t_0_ v, t_0_ v, t_0_* -60901

Note. *z* = starting point, *v* = drift rate, *t_0_* = non-decision time.

As a graphical approach to assess goodness-of-fit, a standard model comparison procedure used in Bayesian parameter estimation — Posterior Predictive Check (PPC) — was performed (Wiecki, Sofer, & Frank, 2013). For the best fitting model, the posterior distributions of the estimated parameters were used to simulate data sets. We then assessed the quality of model fit by plotting the observed data against the simulated data for the .1, .3, .5, .7, and .9 response-time quantiles for each experimental condition (Falbén et al., 2019; Krypotos, Beckers, Kindt, & Wagenmakers, 2015). As indicated in Figure S1, with nearly complete overlap between the observed values and simulated estimates across all prime-target combinations, this revealed good model fit.


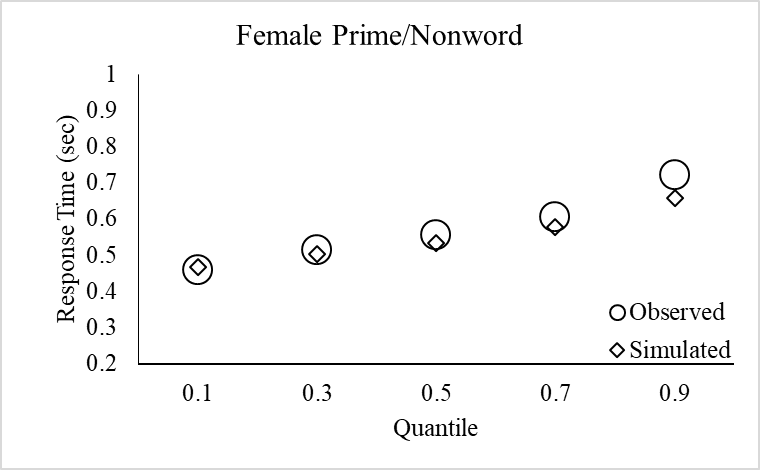

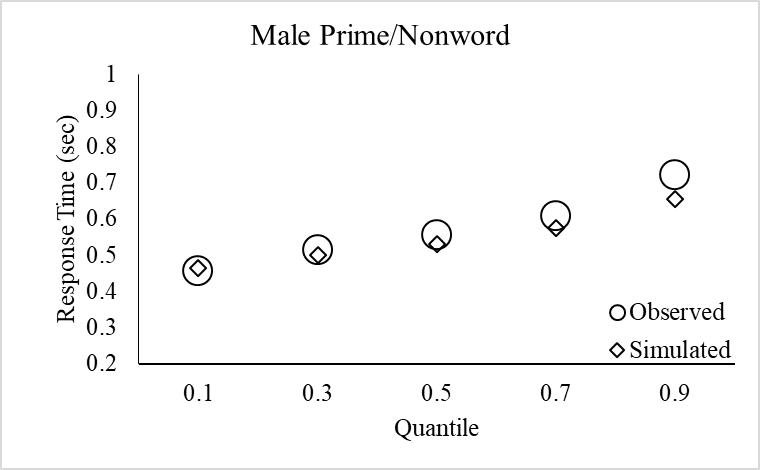

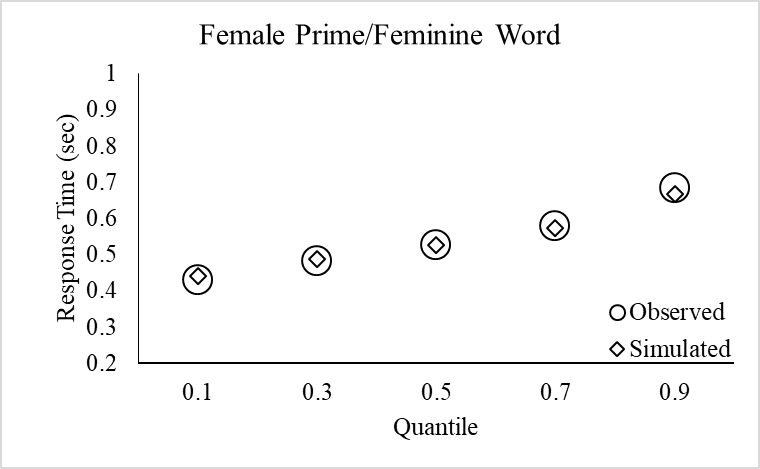

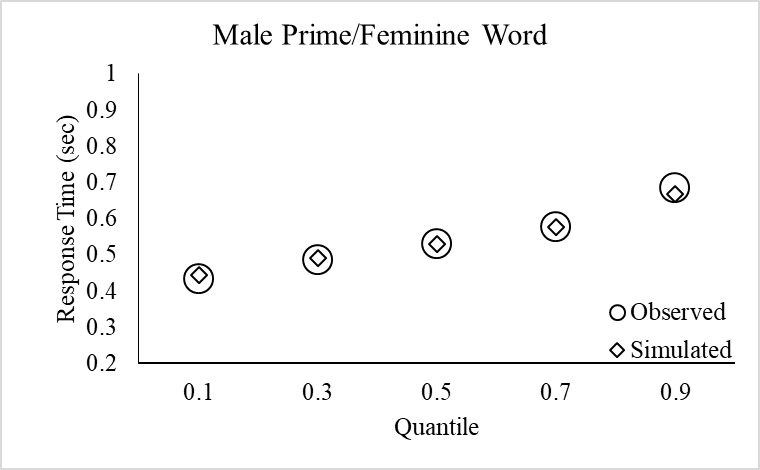


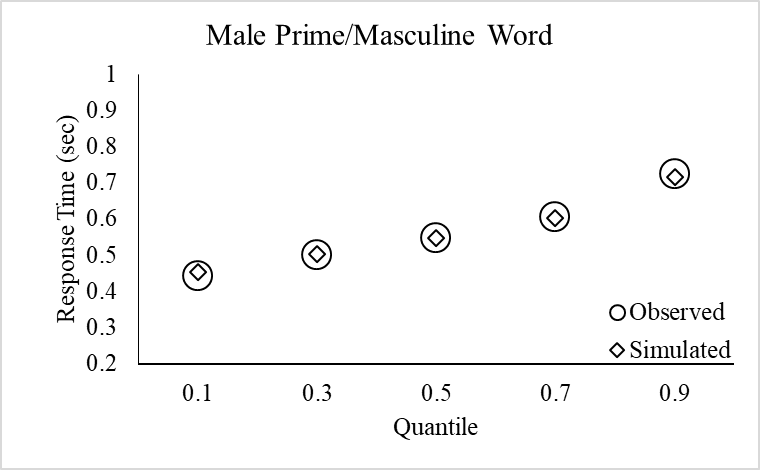

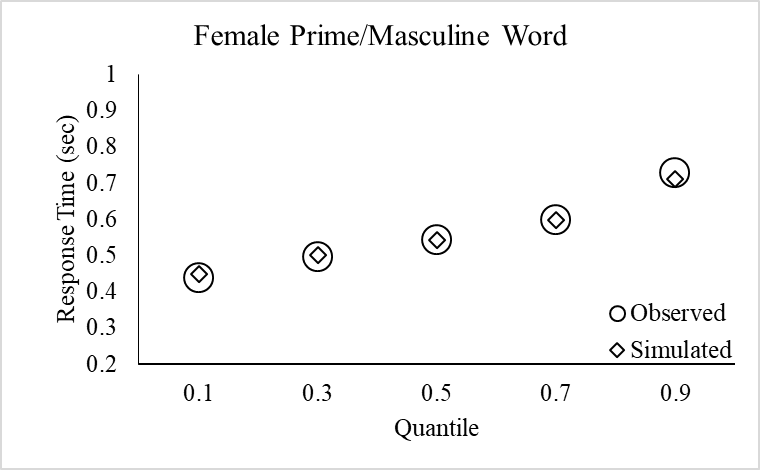


*Figure S1.* Posterior Predictive Check. Comparison of simulated data generated by the best fitting model (i.e., model 6) and the observed data for each experimental condition for the .1, .3, .5, .7, and .9 RT quantiles.

Interrogation of the posterior distributions for model 6 revealed that task performance was underpinned by a combination of response and stimulus biases (see Table S2). Comparison of the observed starting values (female prime: *z* = .51; male prime: *z* = .51) with no bias (*z* = .50) yielded suggestive evidence that less information was required when making word than nonword responses, following both female (*p*_Bayes_[bias > .50] = .120) and male (*p*_Bayes_[bias > .50] = .083) primes. In addition, strong evidence for a stimulus bias was also observed, indicating that information uptake was faster for feminine compared to masculine target words (*p*_Bayes_[feminine word > masculine word] < .001). No differences were observed in the speed of non-decisional processes (*t_0_*).

*Table S2.* Parameter means and 95% Highest Density Intervals (HDI) of the best fitting model for the LDT.

95% HDI

Model Parameter Mean Lower Threshold Upper Threshold

*a* 1.114 1.055 1.176

*s_t0_* 0.132 0.129 0.136

*s_v_* 0.917 0.781 1.047

*s_z_* 0.513 0.474 0.550

*t_0_* _Female Face/Nonword_ 0.423 0.411 0.436

*t_0_* _Male Face/Nonword_  0.439 0.427 0.452

*t_0_* _Female Face/Feminine Word_ 0.423 0.411 0.436

*t_0_* _Female Face/Masculine Word_ 0.426 0.413 0.438

*t_0_* _Male Face/Feminine Word_ 0.426 0.413 0.438

*t_0_* _Male Face/Masculine Word_ 0.428 0.416 0.441

*v* _Nonword_  -4.223 -4.522 -3.927

*v* _Feminine Word_  4.532 4.210 4.860

*v* _Masculine Word_  3.714 3.401 4.025

*z* _Female Face_ 0.510 0.493 0.528

*z* _Male Face_ 0.512 0.495 0.530

Note. *z* = starting point, *v* = drift rate, *a* = boundary separation, *t_0_* = non-decision time, *s_v_* = inter-trial variability of drift rate, *s_z_* = inter-trial variability of starting point, *s_t0_* = inter-trial variability in non-decision time.

**References**

Falbén, J. K., Tsamadi, D., Golubickis, M., Olivier, J. L., Persson, L. M., Cunningham, W. A., & Macrae, C. N. (2019). Predictably confirmatory: The influence of stereotypes during decisional processing. *Quarterly Journal of Experimental Psychology*, *72*(10), 2437–2451.

Krypotos, A-M., Beckers, T., Kindt, M., & Wagenmakers, E-J. (2015). A Bayesian hierarchical diffusion model decomposition of performance in approach-avoidance tasks. *Cognition and Emotion*, *29*, 1424-1444.

Ratcliff, R., & Tuerlinckx, F. (2002). Estimating parameters of the diffusion model: Approaches dealing with contaminant reaction times and parameter variability. *Psychonomic Bulletin & Review*, *9*, 438-481.

Voss, A., Nagler, M., & Lerche, V. (2013). Diffusion models in experimental psychology: a practical introduction. *Journal of Experimental Psychology, 60*(6), 385-402.

Wiecki, T. V., Sofer, I., & Frank, M. J. (2013). HDDM: hierarchical Bayesian estimation of the drift-diffusion model in Python. *Frontiers in Neuroinformatics*, *7*, 14. http://doi.org/10.3389/fninf.2013.00014.
